# Neoadjuvant Immunotherapy in the Treatment of Renal Cell Carcinoma: A Case Series

**DOI:** 10.7759/cureus.27019

**Published:** 2022-07-19

**Authors:** Kevin J Hess, Soraya Bascoy, Ushma Vadher, John Nawrocki, Dhaval Shah

**Affiliations:** 1 Internal Medicine, ChristianaCare, Newark, USA; 2 Internal Medicine-Pediatrics, ChristianaCare, Newark, USA; 3 Oncology, ChristianaCare, Newark, USA

**Keywords:** treatment choices, total neoadjuvant treatment, clear renal cell carcinoma, cancer-immunotherapy, renal cell carcinoma (rcc)

## Abstract

Renal cell carcinoma is a type of urologic cancer that has a poor prognosis, with the majority of these being clear cell renal carcinoma. This subset has a tendency to cause disruptions in the cell cycle, making immune checkpoint inhibitors for adjuvant treatment of renal cell carcinoma the predominant pharmacological approach. Despite this, the use of immune checkpoint inhibitors in this setting is still an area of much research. In the following three different cases, we demonstrate the role and benefit of treatment with neoadjuvant immune checkpoint inhibitors in patients that have an extensive tumor burden at diagnosis, making them ineligible for operative treatment. Our hope is that these cases serve as a foreshadowing of the potential neoadjuvant treatments have in this oncological setting.

## Introduction

Renal cell carcinoma (RCC) is a type of urologic cancer that has a poor prognosis, with 30% of patients having the metastatic disease at the time of diagnosis. About 75% of all renal cell carcinomas are classified as clear cell renal cell carcinoma (ccRCC). Papillary (20%) and chromophobe RCC (5%) represent a much smaller fraction of all diagnosed RCC; translocation-associated RCC, medullary RCC, and collecting duct carcinomas are even rarer forms of this malignancy [1-2]. In its earlier iterations, first-generation immunotherapy employed interleukins or interferons with poor impact on both morbidity and mortality and, as such, innovations in pharmacological approaches to RCC have been the focus of much research in this setting [3-4]. The development of tyrosine kinase inhibitors, mainly vascular endothelial growth factor (VEGF) receptor inhibitors, largely improved progression-free survival and overall survival [4-6]. The emergence of immune checkpoint inhibitors (ICI) alone or in combination with other treatments has shown promising results for treating ccRCCs [7-8]. These immune checkpoint inhibitors tend to target cytotoxic T-lymphocyte antigen-4 (CTLA4) and programmed death protein 1 molecules (PD-1) [4-7]. When these proteins are functional, they result in apoptosis and termination of cytotoxic T-cells that would otherwise provide an additional line of defense in the process of cancer cell destruction. These targeted immunotherapies, which were initially proposed as alternatives to antiangiogenics in the context of ccRCC, later became the mainstay of pharmacological treatment, as these cancers often are typified by high quantities of tumor-infiltrating lymphocytes [4-8]. Current guidelines recommend adjuvant immunotherapy after resection of the primary renal cell carcinoma, however, there is no consistent guidance regarding its use as neoadjuvant immunotherapy [4-8]. The role of these agents as neoadjuvant therapy is thus a potential topic of future research. As such, we use the following case series to demonstrate the potential role of neoadjuvant immunotherapy in renal cell carcinoma treatment.

## Case presentation

Case 1

The first case involves a 53-year-old female with hypothyroidism and hyperlipidemia who was having dyspepsia and epigastric abdominal pain not responsive to a trial of omeprazole. She subsequently underwent an ultrasound of her abdomen, which showed a large right-sided renal mass. A follow-up CT abdomen/pelvis with and without contrast showed a 14.9 cm x 7.5 cm x 7.3 cm renal mass with extension into the renal vein and inferior vena cava, with a concern of extension into the right hepatic lobe. To further stage this, an MRI of the abdomen confirmed the dimensions of this renal mass, as well as re-demonstrated its extension into the aforementioned structures and the posterior right hemidiaphragm. Further imaging of the chest and head for metastatic disease was unremarkable. Biopsy of this mass diagnosed clear cell renal carcinoma; however, given the extent of the tumor, urology considered her a candidate for neoadjuvant treatment prior to any surgical intervention. She was started on pembrolizumab and ipilimumab and after four cycles of therapy, her tumor burden decreased significantly and she was able to undergo a right nephrectomy while remaining on maintenance pembrolizumab every four weeks. On a repeat CT scan of her abdomen/pelvis, she continued to have no evidence of recurrent disease one-year post-resection.

Case 2

This is a 62-year-old gentleman, with no past medical history, who presented to the emergency department with bilateral lower abdominal pain. He also reported increasing urinary frequency, dysuria, nocturia, and fatigue. A CT chest/abdomen/pelvis with IV contrast demonstrated a 10.6 x 10.3 cm left renal mass, renal vein thrombosis, and extension into the inferior vena cava. He also had para-aortic/renal lymphadenopathy, a 12 mm x 15 mm right upper lobe nodule, and a bony lesion at the level of T7 in the thoracic spine. He subsequently underwent a CT-guided biopsy of the left renal mass, which confirmed clear cell renal carcinoma. Just like for our first patient, urology recommended neoadjuvant treatment prior to surgical resection, given the extent of the disease. His treatment course was identical to that of the above case as well; he was started on pembrolizumab and ipilimumab for treatment. After four cycles of therapy, his tumor burden decreased significantly, and he was able to undergo a left nephrectomy while remaining on maintenance pembrolizumab every four weeks. Repeat CT abdomen/pelvis showed post-surgical changes from prior left nephrectomy without any evidence of recurrence one-month post-resection. Two years after his immunotherapy and nephrectomy, he did not show recurrence of his renal cell carcinoma. However, further evaluation of his thoracic mass due to worsening back pain showed a 2.0 x 2.6 cm paraspinal mass at the level of T5/T6, which was ultimately diagnosed as stage II multiple myeloma after serum protein electrophoresis, urine protein electrophoresis, and bone marrow biopsy. He is currently receiving treatment for this diagnosis.

Case 3

Our last case involves a 68-year-old gentleman with type II diabetes complicated by peripheral neuropathy who presented with sudden gross hematuria. On abdominal ultrasound ordered by his primary care provider, he was found to have a large renal mass. This was further elucidated on a CT abdomen/pelvis as a 10.5 cm x 9.0 cm x 12.2 cm renal mass with a concomitant 6 mm hypoattenuating focus in the head of the pancreas. No evidence of metastatic disease was found within the thorax or the brain. CT-guided biopsy of this mass was consistent with clear cell renal carcinoma. Similar to the cases above, he was started on pembrolizumab and ipilimumab. After four cycles of this therapy, followed by maintenance pembrolizumab every four weeks for a total of 16 months of therapy, he ultimately underwent an open partial left nephrectomy. He continues to be free of disease recurrence on repeat CT abdomen/pelvis, with his most recent scan approximately one-year post-surgery.

## Discussion

In the above cases, we provide examples of the utility of neoadjuvant immunotherapy in the treatment algorithm of renal cell carcinoma of the clear cell type. For all of these patients with large, initially inoperable, tumor burden, treatment plans consisted of multiple cycles of immune checkpoint inhibitors targeting PD-1 and CTLA-4 with excellent success. Each of these patients was able to undergo total or partial nephrectomy, followed by maintenance on monotherapy with anti-PD-1 without any recurrence of their primary disease. Motzer et al. noted that targeting multiple sites that help enhance immune response increased overall survival by an additional 13% in comparison to using either anti-PD-1 or anti-CTLA-4 as monotherapy [6-8]. This important principle was demonstrated in each of the above patients treated with ICIs prior to nephrectomy, foreshadowing its benefit as an option for advanced clear cell disease in future studies.

Of note, further optimization of ICI regimens for the treatment of clear cell renal carcinoma is currently ongoing. As of 2020, there were multiple phase III clinical trials evaluating immune checkpoint inhibitors as adjuvant treatment in clear cell renal cell carcinoma. Overall, these trials noted that they had both significantly better objective response rates and complete response rates when compared to the prior first-line therapy of tyrosine kinase inhibitors [9-11]. In the neoadjuvant setting, four studies are evaluating the role of single-agent immunotherapy, one is evaluating dual-agent immunotherapy, and four studies the role of immunotherapy in combination with tyrosine kinase inhibitors or anti-interleukin-1 beta; however, these are currently in progress [11]. Some of these highlighted treatment strategies from an adjuvant treatment regimen have included using pembrolizumab with axitinib, nivolumab with ipilimumab, avelumab with axitinib, and atezolizumab with bevacizumab [7-11]. Unfortunately, when it comes to the treatment of non-ccRCC, the understanding and overall role of ICI is more unclear, and this ultimately remains a further area of opportunity for further studies as well [1,7-11].

## Conclusions

Through this series of cases, we were able to demonstrate the utility of neoadjuvant immune checkpoint inhibitors in the treatment of clear cell renal cell carcinoma. The principles of utilizing anti-PD-1 and anti-CTLA-4-based ICIs in clear cell renal cell carcinoma are being actively studied and have shown improved outcomes from an overall survival standpoint when used in the adjuvant setting. We will ultimately need to see how neoadjuvant studies progress; however, it is our hope that these patients will serve as a representation of what is to further emerge in this rapidly developing area in renal cell carcinoma treatment from the standpoint of morbidity and mortality.
